# Differential expression of pyroptosis-related genes in the hippocampus of patients with Alzheimer’s disease

**DOI:** 10.1186/s12920-023-01479-x

**Published:** 2023-03-14

**Authors:** Pengcheng Xia, Huijun Ma, Jing Chen, Yingchao Liu, Xiaolin Cui, Cuicui Wang, Shuai Zong, Le Wang, Yun Liu, Zhiming Lu

**Affiliations:** 1 Department of Clinical Laboratory Medicine, Shandong Provincial Hospital, Shandong First Medical University, Jinan, Shandong China; 2 Clinical Laboratory, Qingdao Women and Children’s Hospital, Qingdao, Shandong China; 3 Discipline of Anatomy and Pathology, Shandong First Medical University, Jinan, Shandong China; 4 School of Medicine, Shandong University, Jinan, Shandong China

**Keywords:** Alzheimer's disease, Pyroptosis, Hippocampus, Functional enrichment analysis, Protein-protein interaction network

## Abstract

**Background:**

Alzheimer’s disease (AD) is a progressive, neurodegenerative disorder with insidious onset. Some scholars believe that there is a close relationship between pyroptosis and AD. However, studies with evidence supporting this relationship are lacking.

**Materials and methods:**

The microarray data of AD were retrieved from the Gene Expression Omnibus (GEO) database with the datasets merged using the R package inSilicoMerging. R software package Limma was used to perform the differential expression analysis to identify the differentially expressed genes (DEGs). We further performed the enrichment analyses of the DEGs based on Gene Ontology (GO) and the Kyoto Encyclopedia of Genes and Genomes (KEGG) databases to identify the metabolic pathways with a significant difference. The Gene Set Enrichment Analysis (GSEA) was applied to identify the significant pathways. The protein-protein interaction (PPI) network was constructed based on the STRING database with the hub genes identified. Quantitative real-time PCR (qRT-PCR) analyses based on HT22 cells were performed to validate the findings based on the microarray analysis. Gene expression correlation heatmaps were generated to evaluate the relationships among the genes.

**Results:**

A new dataset was derived by merging 4 microarray datasets in the hippocampus of AD patients in the GEO database. Differential gene expression analysis yielded a volcano plot of a total of 20 DEGs (14 up-regulated and 6 down-regulated). GO analysis revealed a group of GO terms with a significant difference, e.g., cytoplasmic vesicle membrane, vesicle membrane, and monocyte chemotaxis. KEGG analysis detected the metabolic pathways with a significant difference, e.g., Rheumatoid arthritis and Fluid shear stress and atherosclerosis. The results of the Gene Set Enrichment Analysis of the microarray data showed that gene set ALZHEIMER_DISEASE and the gene set PYROPTOSIS were both up-regulated. PPI network showed that pyroptosis-related genes were divided into two groups. In the Aβ-induced HT22 cell model, three genes (i.e., *BAX*, *IL18*, and *CYCS*) were revealed with significant differences. Gene expression correlation heatmaps revealed strong correlations between pyroptotic genes and AD-related genes.

**Conclusion:**

The pyroptosis-related genes *BAX*, *IL18*, and *CYCS* were significantly different between AD patients and normal controls.

**Supplementary Information:**

The online version contains supplementary material available at 10.1186/s12920-023-01479-x.

## Introduction

Alzheimer’s disease (AD) is the most common neurodegenerative disease of the elderly, and its incidence increases with age in populations around the world [1]. For example, in 2017, approximately 6.08 million Americans were diagnosed with clinical AD or mild cognitive impairment due to AD, and this number is expected to reach 15 million by 2060.[2]. The most typical clinical manifestation of AD is the gradual decline of cognitive ability in patients, which appears in the early stage of AD without the two pathological signs of senile plaques and neurofibrillary tangles [3, 4]. Although the molecular mechanisms of these two pathological changes have been well studied, therapeutic strategies targeting these changes have not been successful in the treatment of AD. So far, there are no effective drugs to prevent or treat cognitive decline in AD patients.

Pyroptosis, also known as inflammatory necrosis, is a type of programmed cell death characterized by the continuous expansion of cells until the cell membrane ruptures, resulting in the release of cellular contents and the activation of a strong inflammatory response [5]. As a new type of programmed cell death discovered and confirmed in recent years, pyroptosis is characterized by its dependence mainly on caspase-1, caspase-4, caspase-5, and caspase-11 and accompanied by the release of a large number of pro-inflammatory factors [6]. The morphological characteristics, occurrence, and regulatory mechanism of pyroptosis are different from those of other programmed cell death conditions such as apoptosis and necrosis [7]. Pyroptosis mainly relies on the activation of a group of proteins of the caspase family by the inflammasome, cleaving and activating the gasdermin protein, which is translocated to the membrane to form holes and to make cell swell, causing the cytoplasmic outflow and finally leading to cell membrane rupture and pyroptosis [8]. Studies have shown that pyroptosis is widely involved and plays important roles in the occurrence and development of infectious diseases, nervous system-related diseases, and atherosclerotic diseases [9–11]. Pyroptosis also plays important role in AD. For example, studies have shown that amyloid-β induces NOD-like receptor (NLR) family pyrin domain-containing 1 (NLRP1)-dependent neuronal pyroptosis in a mouse model of AD [12], Parkinson disease protein 7 (PARK7/DJ-1) affects oxidative stress and pyroptosis in hippocampal neurons of a mouse model of AD by regulating the nuclear factor-erythroid 2-related factor 2 (Nrf2) pathway [13], while schisandrin inhibits NLRP1 inflammasome-mediated neuronal pyroptosis in a mouse model of AD [14]. To date, the molecular mechanisms regulating the development of pyroptosis are still unclear. Therefore, it is important to identify and investigate the pyroptosis-related genes differentially expressed in AD, to help understand the occurrence of pyroptosis in related diseases, and to further explore their functions in the development, prognosis, and clinical prevention and treatment of these diseases.

In our study, in order to explore the roles of pyroptosis-related genes in AD, microarray datasets from the hippocampus of AD patients were first merged to identify the differentially expressed genes (DEGs) related to AD. The DEGs were further annotated and enriched based on Gene Ontology (GO; http://geneontology.org/) and Kyoto Encyclopedia of Genes and Genomes (KEGG; https://www.genome.jp/kegg/) databases. Through the Gene Set Enrichment Analysis (GSEA) database, the overall gene expression variations associated with AD and pyroptosis were detected. The protein-protein interaction (PPI) network was constructed based on the Search Tool for the Retrieval of Interacting Genes/Proteins (STRING; https://string-db.org/) database to analyze the relationships among the pyroptosis-related genes. The findings were validated with both the dataset GSE48350 and the cellular models of AD, showing that the expressions of genes associated with pyroptosis were significantly altered in AD, providing novel insights into the pathogenesis and potential clinical treatment of AD.

## Materials & methods

### Data preparation

Gene expression profiles of AD were obtained from the Gene Expression Omnibus (GEO) database (https://www.ncbi.nlm.nih.gov/geo/; accessed April 23, 2021). The sample informations involved in all GEO datasets in this study are in Supplementary File S2. The GSE36980 (https://www.ncbi.nlm.nih.gov/bioproject/PRJNA157435) dataset contains gray matter RNA samples from the frontal, temporal cortex, and hippocampus of 88 postmortem brains, 26 of which were pathologically diagnosed AD or AD-like disorder. High-quality RNA (RIN ≥ 6.9) samples were subjected to microarray analysis using the Affymetrix Human Gene 1.0 ST platform, and only results that passed the Human Gene 1.0 ST array quality control check were retrieved. In total, gene expression profiles were collected from three sets of samples: 33 frontal cortex samples (15 from AD patients), 29 temporal cortex samples (10 AD patients), and 17 hippocampal samples (7 AD patients). In particular, in the dataset GSE1297 (https://www.ncbi.nlm.nih.gov/bioproject/PRJNA90219), we have analyzed 9 controls and 22 different severities based on 31 independent microarrays of Hippocampal gene expression in AD subjects and correlation of these gene expressions with MiniMental Status Examination (MMSE) and neurofibrillary tangles (NFT) scores in all 31 subjects. In dataset GSE28146 (https://www.ncbi.nlm.nih.gov/bioproject/PRJNA139561), where the major white matter tracts have been excluded using laser capture microdissection, we extracted formalin from the same subjects’ CA1 hippocampal gray matter was selectively collected from fixed, paraffin-embedded (FFPE) hippocampal sections. The samples in GSE29378 (https://www.ncbi.nlm.nih.gov/bioproject/PRJNA140105) were based on total RNA from 60 μm frozen human hippocampal sections. Control and AD brains were well matched for all non-disease characteristics. Both CA1 and CA3 sections of the same individual were taken from the same section. Several regional and disease-related comparisons were made. Four datasets (GSE36980, GSE1297, GSE28146, and GSE29378) were combined for deg detection in the hippocampus. The dataset GSE 48,350 (https://www.ncbi.nlm.nih.gov/bioproject/PRJNA209800) contains cases from normal controls (NC; ages 20 to 99 years) and AD. The expression changes of synaptic and immune-related genes were analyzed, and the age-related changes, AD-related changes, and region-specific change patterns of gene expression were investigated. These AD cases were processed concurrently with controls (young and old) in dataset GSE11882, which only contains data from normal controls. The dataset GSE 48,350 was used to validate the DEGs identified in the hippocampus. The final data were obtained by combining multiple datasets using the R package from silicomerging [15] to generate a single data matrix (Table S1) and further processing the data matrix using the method of Johnson et al. [16] to remove the batch effects matrix. (Table S2).

### Differential gene expression analysis

Limma [17] is a differential quality articulation screening technique in light of summed up straight models. We played out the differential investigation in light of the R programming bundle Limma (Form 3.40.6) to acquire the DEGs between various examination gatherings and control gatherings. In particular, we originally played out the Log_2_ change of the articulation range dataset and afterward utilized lmFit capability to play out the numerous straight relapse examination. We further utilized eBays capability to compute the directed t-insights, directed f-measurement, and log-chances of differential articulation by observational Bayes balance of the standard blunders towards a typical value, and lastly got the massive distinction of every quality. The changed P-esteem was broken down to address the misleading positive outcomes in the GEO datasets. The boundaries " Adjusted *P* < 0.05 and Log_2_ (Fold Change) > 0.6 or < − 0.6” were characterized as the edges for the screening of differential articulation of mRNAs. The crate plot and heatmap were produced by the capabilities ggplot2 and heatmap, separately, of the R programming bundle.

### GO annotation and KEGG pathway enrichment analysis

We played out the improvement investigations of the DEGs distinguished by Limma in light of KEGG [18] rest Programming interface (https://www.kegg.jp/kegg/rest/keggapi.html) to recognize the metabolic pathways enhanced with massive distinction. The GO comments of the DEGs were performed in view of the R bundle org.hs.eg.db (Version 3.1.0) as the foundation and the R programming bundle clusterProfiler (Version 3.14.3) to acquire the quality sets enhanced with tremendous contrast in light of *P* < 0.05 and false discovery rate (FDR) < 0.25. The base and most extreme qualities were set to 5 and 5000, separately.

### Gene set enrichment analysis

This GSEA (https://www.gseamsigdb.org/gsea/index.jsp) is commonly used to determine statistically significant differences between two biological states (e.g., phenotypes) in an innately defined set of genes [19]. In our study, GSEA was applied to identify important pathways in the merged datasets. The Spearman correlation coefficient between genes and sample labels is defined as the weight of genes [20]. Statistical significance was assessed by comparing the enrichment scores to the enrichment results generated by random permutation of 1000 gene sets to obtain nominal *P*-values. The significance level of metabolic pathways was determined by normalized enrichment score (NES) ≥1.0, FDR ≤ 0.25, *P* ≤ 0.05.

### Protein-protein Interaction (PPI) analysis

A PPI network based on protein-protein interaction (PPI) analysis was established in the STRING database (Version 11.0; http://string-db.org/) [21].

### Validation of pyroptosis-related genes

Validation of the pyroptosis-related genes identified in the microarray datasets was performed based on dataset GSE48350. The pyroptosis-related genes in GSE48350 were compared using the Wilcoxon test. A total of five datasets (i.e., GSE1297, GSE28146, GSE29378, GSE36980, and GSE48350) were used to investigate the association between pyroptosis-related genes and AD-related genes. The two-gene and multiple-gene correlation maps were generated by the R software packages ggstatsplot heatmap, respectively. Spearman’s correlation analysis was performed to analyze the correlations between quantitative variables without normal distributions with the significant difference set to *P* < 0.05. The quantitative real-time PCR (qRT-PCR) analysis was performed using the mouse hippocampal neuron cell line HT22 as the validated cell model induced by 10 µM Aβ1–42 (P9001, rPeptide, Beyotime, Beijing, China) to verify the expression patterns of pyroptosis-related genes revealed in the microarray analysis. The primer sequences were synthesized by RIBOBIO Corporation, Guangzhou, China (File S1). Trizol (Thermo Fisher Scientific Inc., MA, USA) method was employed to extract the total RNA from HT22 cells in each group according to the manufacturer’s protocol. The total RNA (1 µg) was reverse-transcribed to cDNA by use of PrimeScript RT Reagent Kit with gDNA Eraser (Accurate Biotechnology Co., Ltd., Hunan, China). All genes involved in the experiment were examined by a quantitative real-time PCR amplifier (Applied Biosystems QuantStudio 5, ABI Company, Oyster Bay, New York, USA) with SYBR® Premix Ex Taq (Accurate Biotechnology Co., Ltd., Hunan, China). PCR procedure: pre-denaturation at 95 °C for 30 s, 40 cycles of denaturation at 95 °C for 5 s, annealing at 60 °C for 30 s, extension at 72 °C for 30 s, and finally melting at 95 °C for 30 s.

### Statistical analysis

Statistical analysis was performed using GraphPad Prism (version 8.0.0). The data of the GEO dataset were tested for normality and homogeneity of variance. Data that passed these two tests were compared between the two groups using the t-test. *P* values less than 0.05 were considered statistically significant.

## Results

### Analysis of differentially expressed genes (DEGs) in the combined datasets

**Fig. 1:**
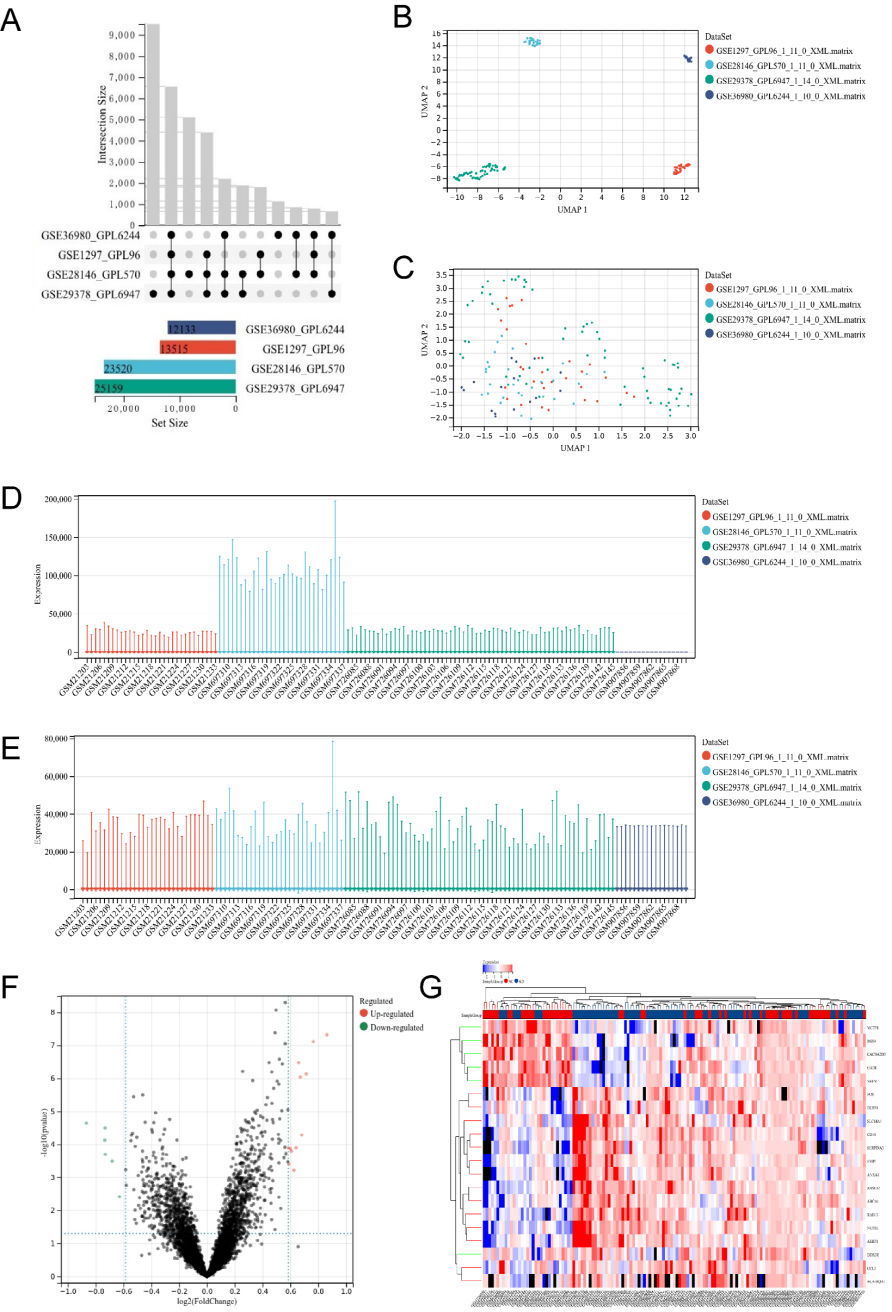
**Analysis of differentially expressed genes (DEGs) based on combined datasets. (A)** Characteristics of combined datasets. **(B)** The Uniform Manifold Approximation and Projection (UMAP) plot before dataset merging. **(C)** UMAP plot after dataset merging. **(D)** Relative expression levels of genes before dataset merging. **(E)** Relative expression levels of genes after dataset merging. **(F)** Volcano plot of DEGs after dataset merging. **(G)** Heatmap of DEGs after dataset merging

Firstly, we merged four gene sets (Table S1) based on the number of genes in the dataset (Fig. 1A). And then, we remove the batch effect between these gene sets. The Uniform Manifold Approximation and Projection (UMAP) plot showed these changes before and after removal (Fig. 1B, C). These are also displayed by the boxplot which showed that the data distributions between the datasets become much more consistent, i.e., the medians existed along the same line (Fig. 1D, E; Table S2). Next, the dataset after the batch effect removal was executed for the differential gene analysis. The volcano plot showed that a total of 20 DEGs existed, including 14 up-regulated and 6 down-regulated (Table 1; Fig. 1F). Their relative expression levels between samples are displayed in the cluster map and heatmap in Fig. 1G.

**Table 1:** The 20 differentially expressed genes (DEGs) were identified in patients with Alzheimer’s disease (AD).

Gene	Log_2_(Fold Change)	*P*-Value		Adjusted P-Value	Regulation	Reported By
*GAD1*	–0.8673		2.26E-05	0.0033	Down	Li S, et al. [22]
*RGS4*	–0.7349		7.44E-05	0.0062	Down	Muma N, et al. [23]
*MCTP1*	–0.7328		3.19E-05	0.0041	Down	Kim K, et al. [24]
*DDX3Y*	–0.7314		0.0002	0.0109	Down	Vakilian H, et al. [25]
*NEFM*	–0.6817		0.0003	0.0145	Down	George C, et al. [26]
*CACNA2D3*	–0.6288		0.0038	0.0523	Down	Huang C, et al. [27]
*RAB13*	0.5865		0.0001	0.0088	Up	Zhang X, et al. [28]
*ABCA1*	0.5894		0.0003	0.0159	Up	Wahrle S, et al. [29]
*DUSP1*	0.5997		0.0001	0.0090	Up	Leandro G, et al. [30]
*AEBP1*	0.6072		0.0001	0.0097	Up	Piras I, et al. [31]
*CCL2*	0.6249		0.0006	0.0212	Up	Hartlage-Rübsamen M, et al. [32]
*ANXA1*	0.6404		0.0001	0.0088	Up	McArthur S, et al. [33]
*NUPR1*	0.6592		3.34E-07	0.0002	Up	Montero-Calle A, et al. [34]
*SLC14A1*	0.6703		9.07E-07	0.0004	Up	Recabarren D, et al. [35]
*SERPINA3*	0.6812		5.21E-05	0.0051	Up	Norton E, et al. [36]
*RASL12*	0.7137		7.37E-07	0.0003	Up	Mirza Z, et al. [37]
*EMP1*	0.7642		7.62E-08	9.73E-05	Up	Ghani M, et al. [38]
*CD44*	0.8621		4.77E-08	7.82E-05	Up	Uberti D, et al. [39]
*HLA-DQA1*	1.0364		0.0012	0.0300	Up	Zhang X, et al. [40]
*FOS*	1.0745		2.35E-05	0.0033	Up	Choi H, et al. [41]

### GO annotation and KEGG pathway enrichment analysis

**Fig. 2:**
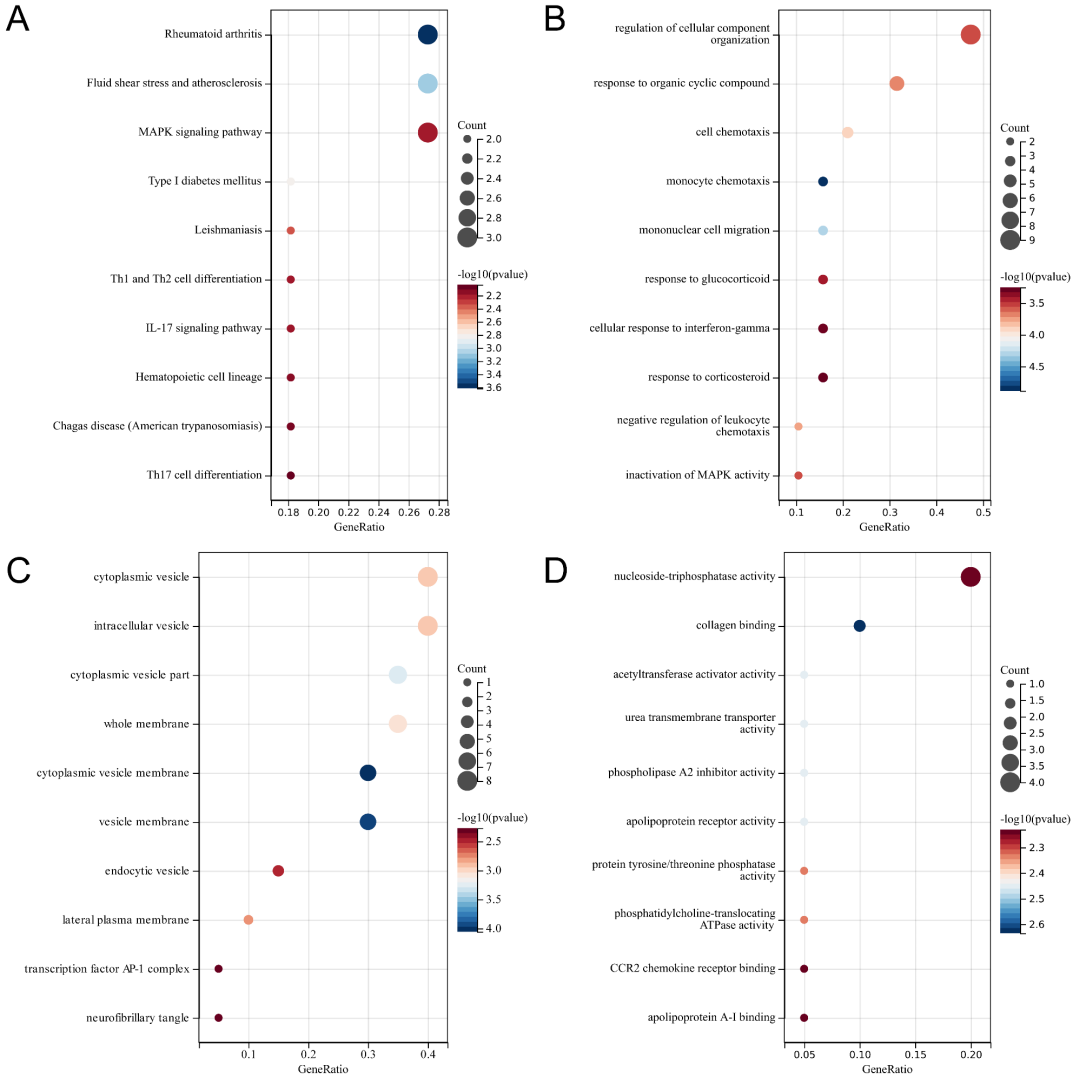
**KEGG and GO analysis of differentially expressed genes (DEGs). (A)** KEGG analysis of DEGs. **(B)** Biological Process (BP) terms of GO analysis of DEGs. **(C)** Cellular Component (CC) terms of GO analysis of DEGs. **(D)** Molecular Function (MF) terms of GO analysis of DEGs.

To further explore the functions and relevant pathways of the potential target genes, the DEGs were analyzed by functional enrichment analyses and KEGG analysis (Fig. 2; Table S3). GO analysis revealed these DEGs mainly focused on the functions such as cytoplasmic vesicle membrane, vesicle membrane, monocyte chemotaxis, cytoplasmic vesicle part, whole membrane, cytoplasmic vesicle, intracellular vesicle, mononuclear cell migration, lateral plasma membrane, and cell chemotaxis. KEGG analysis revealed the following metabolic pathways with a significant difference: Rheumatoid arthritis, Fluid shear stress and atherosclerosis, Type I diabetes mellitus, Leishmaniasis, MAPK signaling pathway, Th1 and Th2 cell differentiation, IL-17 signaling pathway, Hematopoietic cell lineage, Chagas disease (American trypanosomiasis), Th17 cell differentiation, TNF signaling pathway, and Yersinia infection. These results may indicate that immunity, inflammation, and metabolic abnormalities could participate in the occurrence and progress of AD.

### Gene Set Enrichment Analysis (GSEA)

**Fig. 3:**
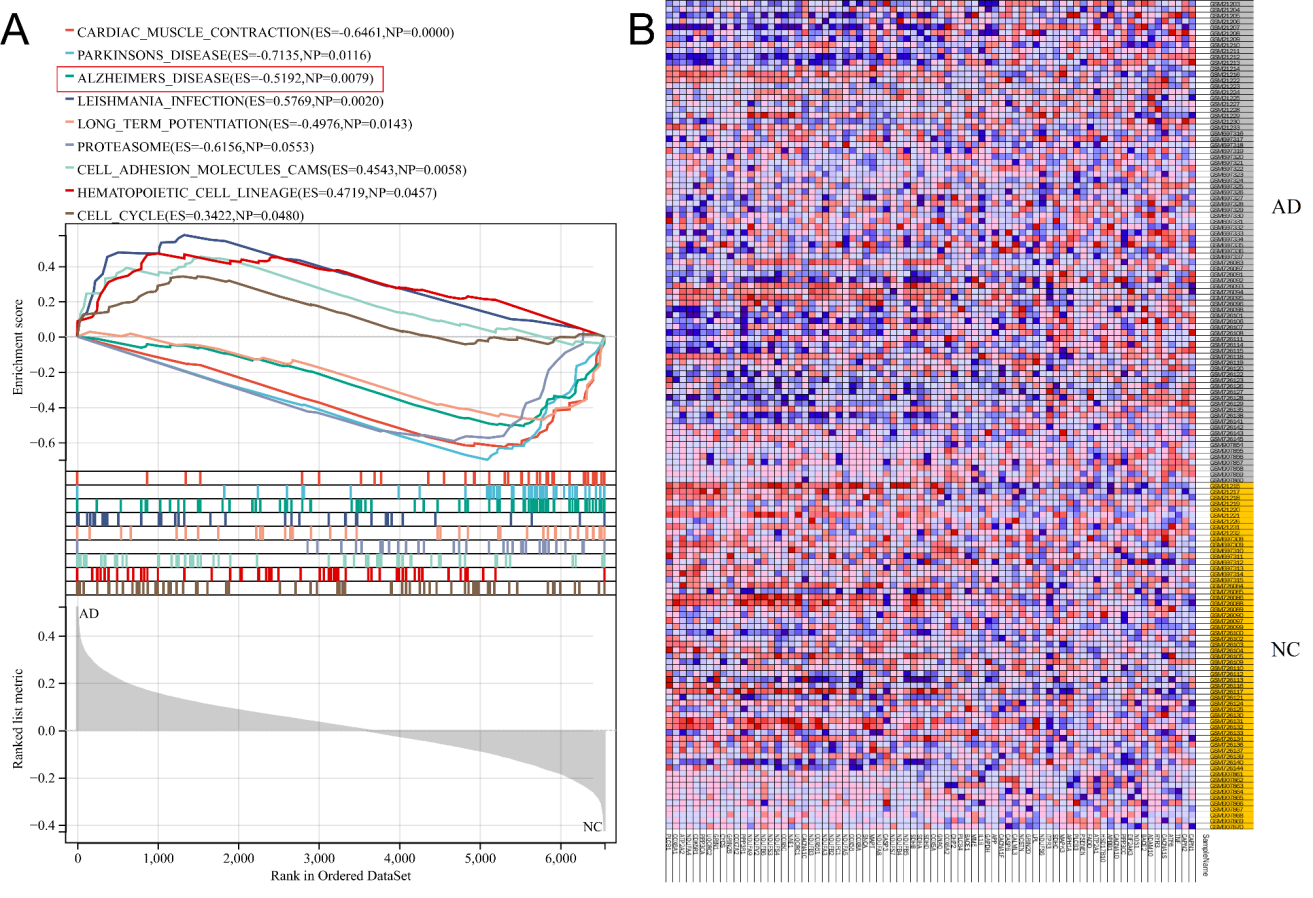
**GSEA analysis of merged datasets. (A)** Differentially expressed gene (DEG) sets between Alzheimer’s disease (AD) and NC groups. **(B)** Heatmap of AD gene sets between AD and NC groups

**Fig. 4:**
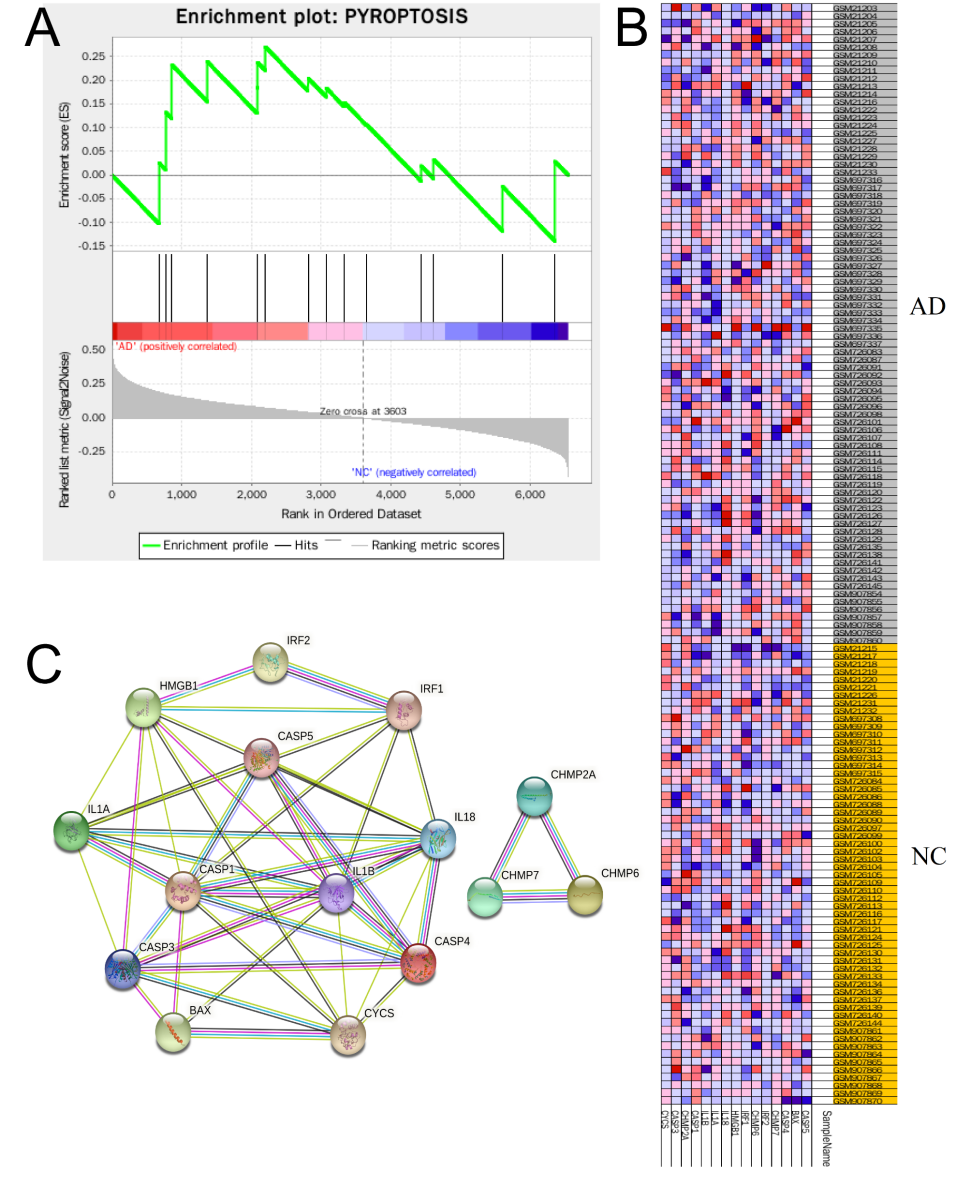
**GSEA analysis and protein-protein interaction (PPI) network of the pyroptotic gene set. (A)** GSEA analysis of the pyroptotic gene set. **(B)** Heatmap of the pyroptotic gene set. **(C)** PPI network of the pyroptotic gene set

To explore the merged hippocampal dataset more comprehensively, we conducted the GSEA. The results showed that the gene set ALZHEIMER_DISEASE was higher expressed in the AD group than in the NC group (Fig. 3A, B; Table S4). The relative expression levels of the representative genes between the samples were shown in Fig. 3A. Considering the DEGs enriched in inflammation-related pathways, we carried out GSEA analysis based on the gene set PYROPTOSIS. Interestingly, the gene set ALZHEIMER_DISEASE and PYROPTOSIS from the merged hippocampal dataset showed similar expression trends(Fig. 4A, B; Table S5), implying that pyroptosis-related genes may participate in the occurrence of AD.

### PPI network construction

Subsequently, all the pyroptosis-related genes were further analyzed by the STRING database to construct the PPI network (Fig. 4C; Table. S6). A total of 12 nod genes (i.e., *CASP5*, *BAX*, *CASP4*, *IRF2*, *IRF1*, *HMGB1*, *IL18*, *IL1A*, *IL1B*, *CASP1*, *CASP3*, and *CYCS*) were revealed in the same collective, while another three nod genes (i.e., *CHMP2A, CHMP6*, and *CHMP7*) were defined in another collective.

### Correlation between pyroptosis-related genes and AD-related genes

**Fig. 5:**
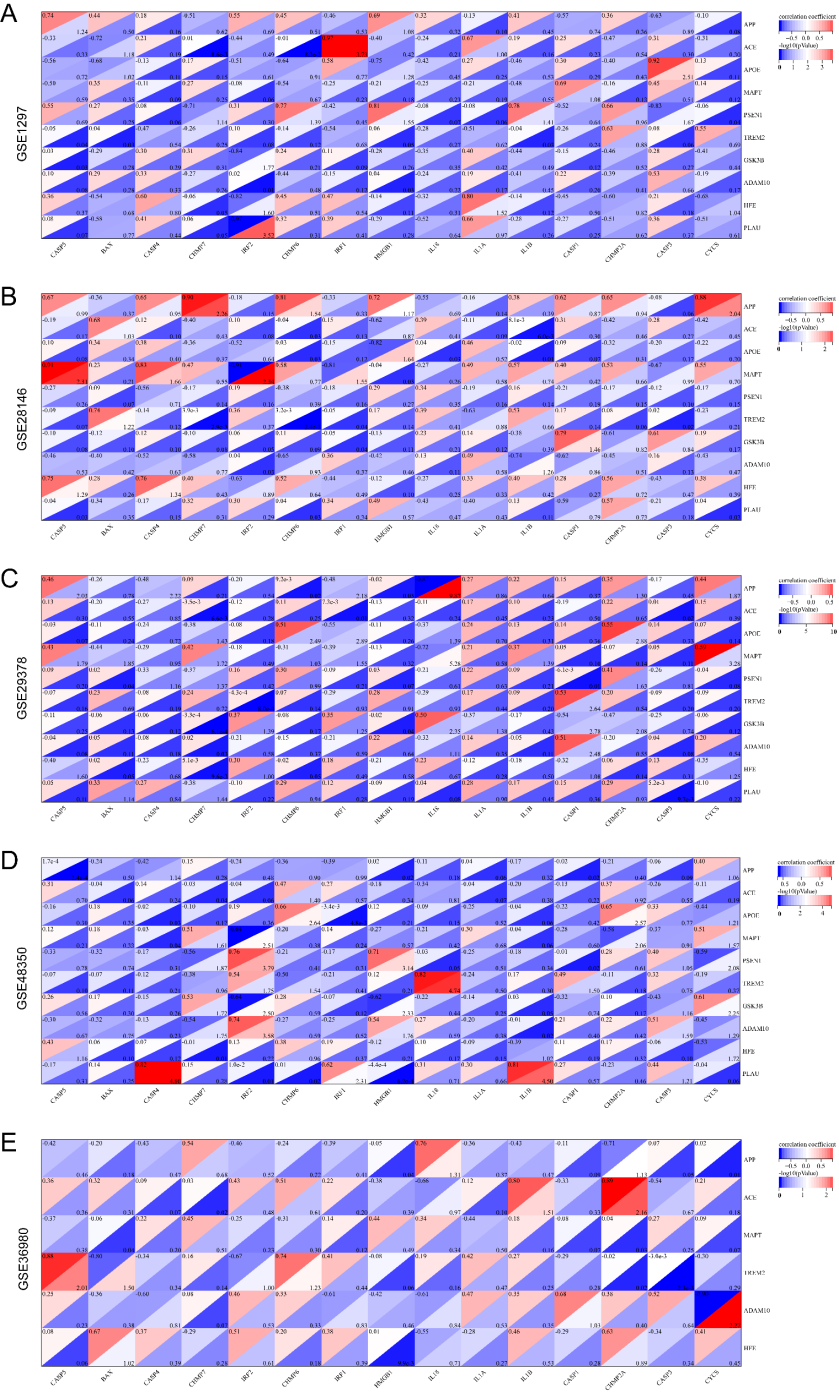
**Heatmaps of the correlation between Alzheimer’s disease (AD)-related genes and pyroptosis**-**related genes of datasets GSE1297 (A), GSE28146 (B), GSE29378 (C), GSE48350 (D), and GSE36980 (E).** The abscissa and ordinate represent genes. Different colors represent different correlation coefficients, i.e., red for positive correlation and blue for negative correlation, with the darker color representing the stronger correlation

Then we carried out the correlation exploration between pyroptosis-related genes from GSEA and AD-related genes from Disgenet in the datasets (Table S7). A large number of genes between them showed significant correlations. *IRF1* was most positively correlated with *ACE* in GSE1297 (Fig. 5A), *IRF2* was most negatively correlated with *MAPT* in GSE28146(Fig. 5B), *IL18* was most negatively correlated with *APP* in GSE29378 (Fig. 5C), *CASP4* was most positively correlated with *PLAU* in GSE48350(Fig. 5D), *CYCS* was most negatively correlated with *ADAM10* in GSE36980 (Fig. 5E). Notably, GSE36980 contained only 6 of the top 10 genes associated with AD due to the analyses based on different platforms.

### Validation of pyroptosis-related genes

**Fig. 6:**
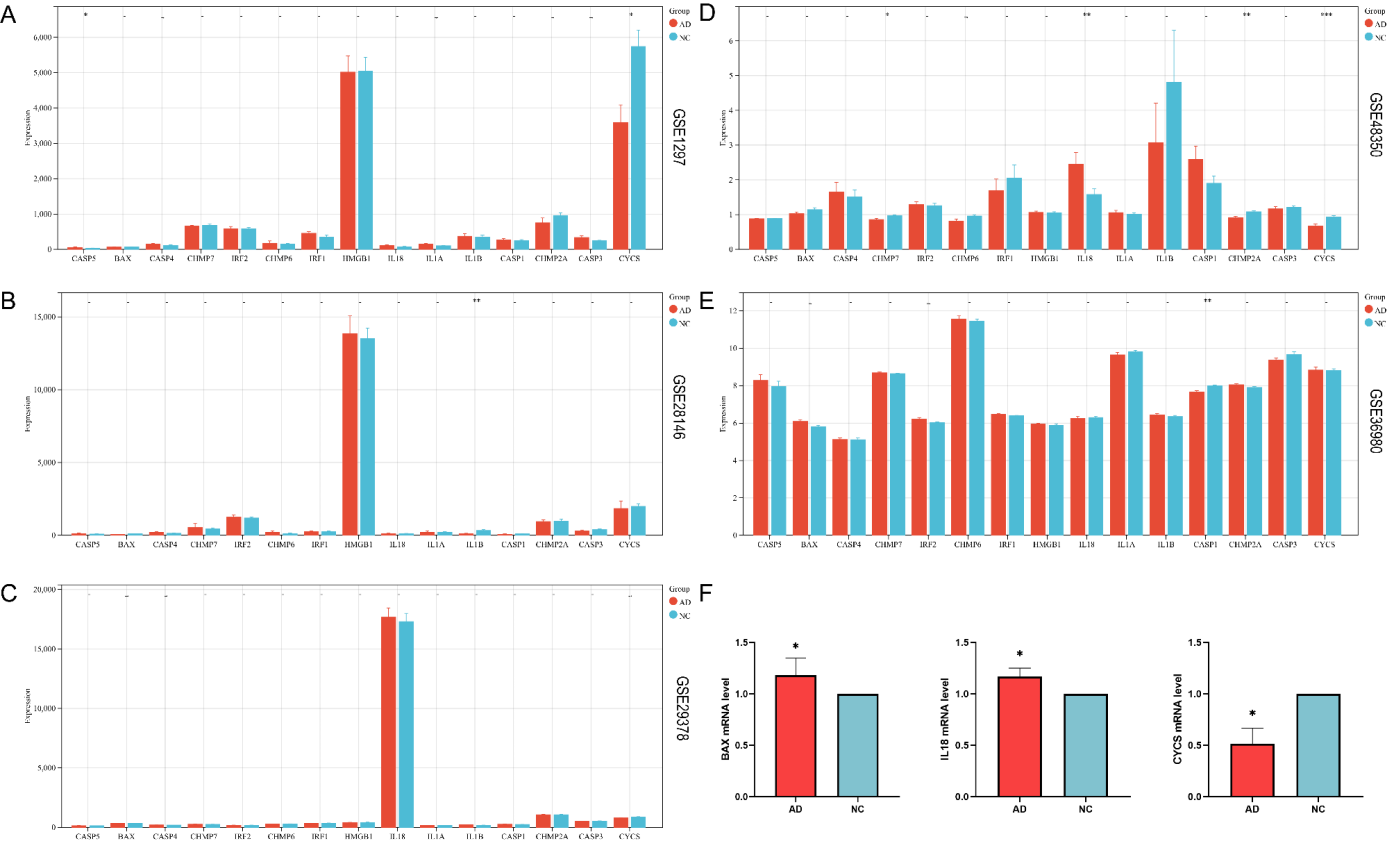
Expression levels of pyroptosis-related differentially expressed genes (DEGs) in five datasets of GSE1297 (A), GSE28146 (B), GSE 29,378 (C), GSE48350 (D), and GSE36980 (E), and the HT22 cell models of Alzheimer’s disease (AD) (F)

Lastly, the expression levels of the pyroptosis-related genes identified in the dataset were further evaluated. Compared with the NC group, the expression of *CASP5* (Fig. 6A) and *IL18* (Fig. 6D) in the AD group was increased in GSE1297 and GSE48350 respectively, while the expression of *CYCS* (Fig. 6A), *IL1B* (Fig. 6B) and *CASP1*(Fig. 6E) was decreased in GSE1297, GSE28146, and GSE36980 respectively, and the expressions of *CHMP7*, *CHMP2A*, and *CYCS* were decreased in GSE48350 (Fig. 6D). In GSE29378, pyroptosis-related genes showed no statistical differences (Fig. 6C). In the Aβ-induced HT22 cell model of AD, a total of three genes (i.e., *BAX*, *IL18*, and *CYCS*) showed a significant difference in their expressions (Fig. 6F, Table S8). These results confirmed that pyroptosis-related genes may participate in the occurrence of AD.

## Discussion

Studies have hinted that there might be a possible relationship between pyroptosis and AD development [42–45], however, the experimental evidence supporting this correlation is sparse. In our study, we demonstrated that there was a strong relevance between pyroptosis-related and AD-related genes, and the pyroptosis-related genes were differentially expressed in the hippocampus of AD patients and models, which provided strong experimental evidence to support the involvement of pyroptosis in the development of AD.

The hippocampus is located between the thalamus and the medial temporal lobe of the brain. It is a part of the limbic system, mainly responsible for the storage, conversion, and orientation of short-term memory, and also confirmed to play an important role in the development of AD [46]. Thus, we explored four hippocampal sequencing datasets of AD patients and found 20 DEGs between healthy people and AD patients. The previous research proved that most of them were involved in nervous diseases, for example, *GAD1* was involved in the neuropathology of schizophrenia [21], RGS4 showed decreased mRNA levels in the prefrontal cortex from AD patient autopsies [47], *CD44* was increased in lymphocytes derived from AD patients [39], and the FOS exhibited the intensification of immunoreactivity in AD cases [48]. These DEGs played vital roles in nervous systems, hinting at their possible associations with AD.

Function analysis was performed based on these DEGs to delve into the exact pathway. KEGG models showed most of DEGs enriched in pathways relative to immunity and inflammation, like MAPK, IL-17 signaling pathway, and Th1, Th2, and Th17 cell differentiation, while GO models also showed in inflammation like monocyte chemotaxis, mononuclear cell migration, and so on [49, 50]. A study has shown that the main indications of pyroptosis include the formation of inflammasomes, the activation of caspase and gasdermin, and the release of a large number of pro-inflammatory factors [44]. The typical pathway involved in pyroptosis generally includes the caspase-1 pathway identifying the detrimental effects through the inflammasome, recruiting, activating, and cleaving caspase-1, activating inflammatory factors such as IL-18 and IL-1β, cleaving the N-terminal sequence of gasdermin D (GSDMD) to bind to membranes to create the membrane pores, ultimately leading to pyroptosis [51]. Therefore, we conducted a GSEA analysis of AD-related and pyroptosis-related genes based on the combined dataset. Notably, these two gene sets showed similar trends, indicating that lesions in the hippocampus were closely associated with AD, and the gene set of pyroptosis could participate in this disease. Subsequently, we conducted PPI network construction on a pyroptosis-related gene and correlation analysis between pyroptosis-related genes and AD-related genes. A large number of pyroptosis-related genes and AD-related genes in the datasets showed significant correlations, which confirmed our conjecture that pyroptosis could play a vital role in AD.

It is well-known that inflammasomes play important roles in the development of AD, especially NLRP3 inflammasomes. [52, 53]. And activation of the NLRP3 inflammasome could cause caspase-1-mediated production of interleukin (IL)-1β and IL-18 in microglia [54]. A study reported that fatal epilepsy in IL18 KO/APP/PS1 mice was completely reversed by the anticonvulsant levetiracetam, while the IL18-deficient AD mice with chemically induced seizures exhibited lower thresholds and increased gene expression associated with increased neuronal activity [55], which implied that IL18 might be involved in the development of AD. Our PCR analysis showed that the expressions of *IL18* were increased in the AD model which confirmed this point. Meanwhile, the level of *Bax* was also raised. The research found that the localization of bax in senile plaques in the hippocampi of AD patients was correlated with the localization of the β-amyloid protein in the adjacent sections of the same brain, while bax was generally strongly stained in tau-positive tangles in the AD hippocampi, suggesting its vital role in tangle formation [56, 57]. Furthermore, the levels of bax were decreased in the dentate granule cells of the AD hippocampi, which was probably related to the survival of the neurons in AD [58]. To date, rare studies revealed the functions of CYCS in AD. One paper mentioned its possible value in AD diagnosis, however, the exploration of the mechanism is scarce. It has been reported that CYCS plays a role in apoptosis, while the inhibition of anti-apoptotic members of the BCL-2 family or the activation of pro-apoptotic members could lead to changes in the permeability of the mitochondrial membrane, thereby reducing the release of CYCS into the cytoplasm [59–61]. Our work found that the mRNA levels of *CYCS* decreased in the HT22 cell model of AD, which supported these points in depth.

Taken together, these results demonstrated that pyroptosis played an important role in AD. Further verifications are needed for the DEGs and the molecular mechanisms with the metabolic pathways involved in the development of AD revealed in this study.

## Conclusion

The pyroptosis-related genes *BAX*, *IL18*, and *CYCS* were significantly different between AD patients and normal controls. This proves that the mechanism of pyroptosis is very important for AD, and these significantly differentially expressed genes can be potential targets for the diagnosis and treatment of AD.

## Electronic supplementary material

Below is the link to the electronic supplementary material.

Supplementary Material 1

Supplementary Material 2

Supplementary Material 3

Supplementary Material 4

Supplementary Material 5

Supplementary Material 6

Supplementary Material 7

Table S8. Primers and their sequences used in the quantitative real-time PCR of the pyroptosis-related genes

Supplementary Material 9

Supplementary Material 10

## Data Availability

The datasets generated and/or analyzed during the current study are available in the Gene Expression Omnibus (GEO) repository [68]. The links to the GEO datasets covered in this article are as follows. GSE1297: https://www.ncbi.nlm.nih.gov/geo/query/acc.cgi?acc=GSE1297; GSE28146: https://www.ncbi.nlm.nih.gov/geo/query/acc.cgi?acc=GSE28146; GSE 29378: https://www.ncbi.nlm.nih.gov/geo/query/acc.cgi?acc=GSE29378; GSE36980: https://www.ncbi.nlm.nih.gov/geo/query/acc.cgi?acc=GSE36980; GSE48350: https://www.ncbi.nlm.nih.gov/geo/query/acc.cgi?acc=GSE48350.

## Abbreviations

AD: Alzheimer’s Disease
DEGs: Differentially Expressed Genes
GEO: Gene Expression Omnibus
GO: Gene Ontology
KEGG: Kyoto Encyclopedia of Genes and Genomes
MEs: Module Eigengenes
MM: Module Membership
PPI: Protein-protein Interaction
NES: Normalized Enrichment Score
FDR: False Discovery Rate
A-β: Amyloid β-protein
GSEA: Gene Set Enrichment Analysis
q-RT-PCR: quantitative real-time PCR
