# Supplementary material for: Differential expression of pyroptosis-related genes in the hippocampus of patients with Alzheimer’s disease
Source: BMC Med Genomics. 2023 Mar 14;16:56. doi: 10.1186/s12920-023-01479-x (PMC10012531; doi:10.1186/s12920-023-01479-x)
Supplement: Supplementary file 8 — Table S8. Primers and their sequences used in the quantitative real-time PCR of the pyroptosis-related genes [file 12920_2023_1479_MOESM8_ESM.docx]

**Table S8. Primers and their sequences used in the quantitative real-time PCR of the pyroptosis-related genes**

| **Gene** | **primer-F (5'-3')** | **primer-R (5'-3')** | **PCR product length (bp)** |
| --- | --- | --- | --- |
| *β-actin* | AACAGTCCGCCTAGAAGCAC | CGTTGACATCCGTAAAGACC | 295 |
| *BAX* | CAGGATGCGTCCACCAAGAA | CGTGTCCACGTCAGCAATCA | 102 |
| *CASP4* | CTTAGGCTACGATGTGGTGGTGAA | AGGAATGTGCTGTCTGATGTCTGG | 111 |
| *CHMP7* | CCCTCAAGTGGACTCTTTCTAACA | GCGAGTTCTGGTATAGGCGATA | 123 |
| *IRF2* | CCAGTGATGAAGAGAACGCAGAG | GCAGATAGGTGTTCCGTGTCC | 102 |
| *CHMP6* | GAGCATCGAGTTCACGCAGAT | CCACCTCCTCTATGGACATCAC | 104 |
| *IRF1* | GTGTCGTCAGCAGCAGTCTCT | TTCGGCTATCTTCCCTTCCTCATC | 149 |
| *HMGB1* | TGGCTTTTGTCCCTCATCCTT | GAGGCCGCAGTTTCCTATCG | 107 |
| *IL18* | TGACCAAGTTCTCTTCGTTGACAA | CACAGCCAGTCCTCTTACTTCAC | 136 |
| *IL1B* | TCCAGGATGAGGACATGAGCAC | GAACGTCACACACCAGCAGGTTA | 105 |
| *IL1A* | CTGAAGAAGAGACGGCTGAGT | CTGGTAGGTGTAAGGTGCTGAT | 114 |
| *CASP1* | ACATCTTTCTCCGAGGGTTGG | GGCAGGCAGCAAATTCTTTCA | 143 |
| *CHMP2A* | TGAGGAAGATGAAGAGGAGAGTGA | CAGTGGAGGGAAGGTTTGACA | 98 |
| *CASP3* | AGAGACATTCATGGGCCTGAAATAC | CACCATGGCTTAGAATCACACACAC | 143 |
| *CYCS* | CCAAATCTCCACGGTCTGTTC | ATCAGGGTATCCTCTCCCCAG | 107 |
